# Early-Onset Thrombocytopenia in Small-For-Gestational-Age Neonates: A Retrospective Cohort Study

**DOI:** 10.1371/journal.pone.0154853

**Published:** 2016-05-13

**Authors:** S. F. Fustolo-Gunnink, R. D. Vlug, V. E. H. J. Smits-Wintjens, E. J. Heckman, A. B. te Pas, K. Fijnvandraat, E. Lopriore

**Affiliations:** 1 Sanquin Blood Supply, Clinical Transfusion Research, Leiden, Zuid-Holland, The Netherlands; 2 Academic Medical Center, Pediatric hematology, Amsterdam, Noord-Holland, The Netherlands; 3 Division of Neonatology, Department of Pediatrics, Leiden University Medical Center, Leiden, The Netherlands; Hôpital Robert Debré, FRANCE

## Abstract

Thrombocytopenia is a common finding in small for gestational age (SGA) neonates and is thought to result from a unique pathophysiologic mechanism related to chronic intrauterine hypoxia. Our objective was to estimate the incidence and severity of early-onset thrombocytopenia in SGA neonates, and to identify risk factors for thrombocytopenia. We performed a retrospective cohort study of all consecutive SGA neonates admitted to our ward and a control group of appropriate for gestational age (AGA) neonates matched for gestational age at birth. Main outcome measures were incidence and severity of thrombocytopenia, hematological and clinical risk factors for thrombocytopenia, and bleeding. A total of 330 SGA and 330 AGA neonates were included, with a mean gestational age at birth of 32.9 ± 4 weeks. Thrombocytopenia (<150x10^9^/L) was found in 53% (176/329) of SGA neonates and 20% (66/330) of AGA neonates (relative risk (RR) 2.7, 95% confidence interval (CI) [2.1, 3.4]). Severe thrombocytopenia (21-50x10^9^/L) occurred in 25 neonates (8%) in the SGA and 2 neonates (1%) in the AGA group (RR 12.5, 95% CI [3.0, 52.5]). Platelet counts <20x10^9^/L were not recorded. Within the SGA group, lower gestational age at birth (p = <0.01) and erythroblastosis (p<0.01) were independently associated with a decrease in platelet count. Platelet count was positively correlated with birth weight centiles. In conclusion, early-onset thrombocytopenia is present in over 50% of SGA neonates and occurs 2.7 times as often as in AGA neonates. Thrombocytopenia is seldom severe and is independently associated with lower gestational age at birth and erythroblastosis.

## Introduction

Thrombocytopenia, defined as a platelet count below 150×10^9^/L, occurs in 1 to 5% of all neonates and in 22 to 35% of neonates admitted to a neonatal intensive care (NICU).[1–7] The causes of neonatal thrombocytopenia vary according to the underlying disease and can be classified according to the time at onset. Early-onset thrombocytopenia occurs within 3 days after birth, whereas late-onset thrombocytopenia occurs more than 3 days after birth. In general, early onset thrombocytopenia is associated with prenatal factors, such as maternal disease or placenta insufficiency, whereas late onset thrombocytopenia is often caused by sepsis or necrotizing enterocolitis. One of the most frequent causes of early-onset thrombocytopenia is intrauterine growth restriction and it is therefore often detected in small for gestational age (SGA) neonates.[2–4,7]

In SGA neonates, early-onset thrombocytopenia is thought to result from increased platelet consumption in the placenta due to increased blood flow resistance and/or result from suppressed platelet production due to increased red cell production.[8,9] Not all SGA neonates develop thrombocytopenia, and severity varies, therefore other factors are thought to play an etiological role as well. Several studies on hematologic parameters in SGA neonates have been published.[7,10–16] However, information about risk factors for thrombocytopenia specifically within the SGA group is scarce.

The aims of this study are to estimate the incidence and severity of thrombocytopenia in SGA neonates compared to a matched appropriate for gestational age (AGA) control group, to assess risk factors for thrombocytopenia within the SGA group, and to describe the occurrence of bleeding in both groups.

## Methods

### Population

All SGA neonates admitted to the NICU of the Leiden University Medical Center (LUMC) between January 2007 and February 2012 were included. The LUMC is one of 10 tertiary neonatal care centers in the Netherlands. We excluded neonates with fetal and neonatal alloimmune thrombocytopenia, idiopathic thrombocytopenic purpura and rhesus alloimmunization. Each SGA neonate was matched with the next admitted AGA neonate with a similar gestational age at birth (+/- 1 week). SGA was defined as a birth weight below the 10^th^ centile according to Dutch national birth weight curves.[17] SGA was subdivided into mild SGA (5^th^-10^th^ centile), moderate SGA (2.3^th^-5^th^ centile) and severe SGA (<2.3^th^ centile).

### Data collection

Data were extracted from the LUMC patient database, including medical files and laboratory outcomes.

The following maternal data were extracted: maternal hypertension (defined as a diastolic blood pressure of 90 mmHg or more on two occasions more than 4 hours apart, or a single diastolic blood pressure above 110 mmHg), and mode of delivery. The following neonatal data were extracted: birth weight, gender, occurrence of severe hemorrhage, hematologic parameters, early onset sepsis (defined as a positive blood culture in the first 3 days of life) and neonatal mortality. Apart from mortality, all data were collected within the first 3 days of life. Severe hemorrhage was defined as the presence of severe intraventricular hemorrhage (IVH) (grade 3 or 4, according to Papile), or severe pulmonary or gastrointestinal hemorrhage requiring treatment with platelet transfusions, volume expanders and/or red blood cell transfusions.[18,19] The following hematologic data were recorded; platelet count, hemoglobin level, white blood cell count and nucleated red blood cell (NRBC) count during the first 3 days of life. Hematological investigations are routinely performed at birth in all neonates admitted to our NICU. Thrombocytopenia was defined as a platelet count <150x10^9^/L and was classified as mild (101 to 149x10^9^/L), moderate (51 to 100x10^9^/L), severe (21 to 50x10^9^/L) and very severe (≤20x10^9^/L). We recorded the use of platelet transfusions during the first 3 days of life. According to our transfusion guidelines, a concentrated or hyperconcentrated platelet transfusion in a dose of 20x10^9^/kg was given if: (1) platelet count was <30x10^9^/L (january 2007-november 2009) or <20x10^9^/L (since 2009, due to a protocol change) or (2) platelet count was <50x10^9^/L in neonates with a manifest bleeding/undergoing a procedure with risk of bleeding or in neonates with a birth weight <1500 gram and gestational age < 32 weeks who were clinically ill. Erythroblastosis was adjusted for gestational age and defined as nucleated red blood cells (NRBC)/100 white blood cells (WBC). Leucocytopenia was defined as a white blood cell count below 5x10^9^/L.

### Statistics

All variables were analyzed using the student *t* test for continuous variables, the chi-square test or Fisher’s exact test for categorical variables, and non-parametric testing where appropriate. The following potential predictors for platelet count were studied in a univariate linear regression model: maternal hypertension, gestational age at birth, birth weight, severity of SGA and erythroblastosis. Predictors for platelet count that were significant (p<0.05) in the univariate analysis were included in a multivariate linear regression model. The results of the linear models were expressed as coefficients and 95% confidence intervals (CI). Linear regression was used to evaluate the relationship between birth weight centiles and lowest platelet and NRBC counts in the first three days of life. A p-value <0.05 was considered statistically significant. Statistical analyses were performed using SPSS Statistics 20.0 (SPSS, Chicago, Illinois, USA). Because our study involves anonymized patient data, no medical ethics approval is necessary according to Dutch law.

## Results

The total number of admissions during the study period was 3905. A total of 330 (8.5%) neonates with SGA met the inclusion criteria and were matched with a control group of 330 AGA neonates.

### Baseline characteristics

In the study group of SGA neonates, 167 (51%) had mild SGA, 74 (22%) moderate SGA and 89 (27%) severe SGA. Maternal hypertension and cesarean delivery occurred more often in the SGA group. Further details of the baseline characteristics are presented in Table 1.

**Table 1:** Baseline characteristics.

	SGA (n = 330)		AGA (n = 330)	
Maternal hypertension–n (%)	127	(38%)	82	(25%)
Caesarean delivery–n (%)	208	(63%)	124	(38%)
Female–n (%)	152	(46%)	169	(51%)
Gestational age at birth (weeks)–mean ± SD	32.9	± 4.0	32.9	± 4.0
Birth weight (grams)–median (IQR)	1233	(980)	1913	(1354)

SGA = small for gestational age, birth weight < 10^th^ centile. AGA = appropriate for gestational age, birth weight > 10^th^ centile. IQR = interquartile range

### Hematologic outcome

**Fig 1:**
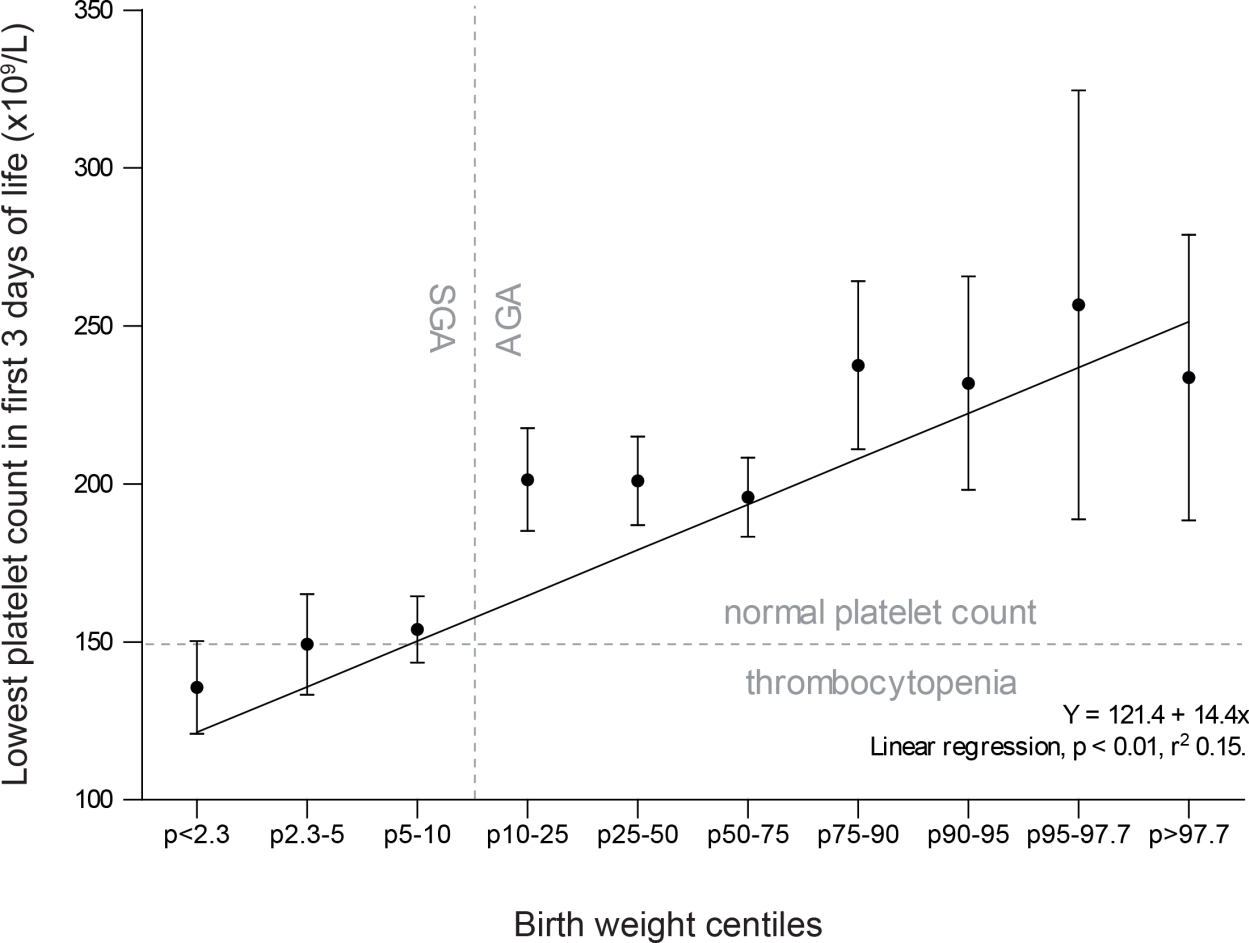
Relationship between lowest platelet counts in the first 3 days of life and birth weight centiles. Boxplot representing observed lowest platelet counts (x10^9^/L) in all included neonates (SGA and AGA, N = 659) in the first 3 days of life, categorized by birth weight centiles. Boxplot shows mean (black dots) and 95% confidence intervals (whiskers), with a regression line.

Platelet counts were measured in all but one neonate at least once within the first 3 days of life. Thrombocytopenia was found in 37% (242/659) of all neonates and was more than twice as frequent in SGA neonates (53%) compared to AGA neonates (20%) (relative risk (RR) 2.7, 95% confidence interval (CI) [2.1, 3.4]). The relationship between birth centiles and platelet count is depicted in Fig 1, and shows a positive correlation between platelet counts and birth weight centile (p<0.01). For every step up in birth weight centile group (e.g. from the ‘10–25’ group to the ‘25–50’ group), platelet count increases with 14x10^9^/L. Severe thrombocytopenia (20-50x10^9^/L) was present in 8% (25/329) of SGA neonates and 1% (2/330) of AGA neonates (RR 12.5, 95% CI [3.0, 52.5]). No cases of very severe thrombocytopenia (<20x10^9^/L) were detected. Of the 27 neonates who developed severe thrombocytopenia, only 5 had severe thrombocytopenia on the first day of life. Five had moderate thrombocytopenia, 10 had mild thrombocytopenia, 2 had no thrombocytopenia, and 5 had no platelet count measured on their first day of life. In the SGA group, 5% of neonates (17/330) received a platelet transfusion, while no neonates in the AGA group required a platelet transfusion (p<0.01).

**Fig 2:**
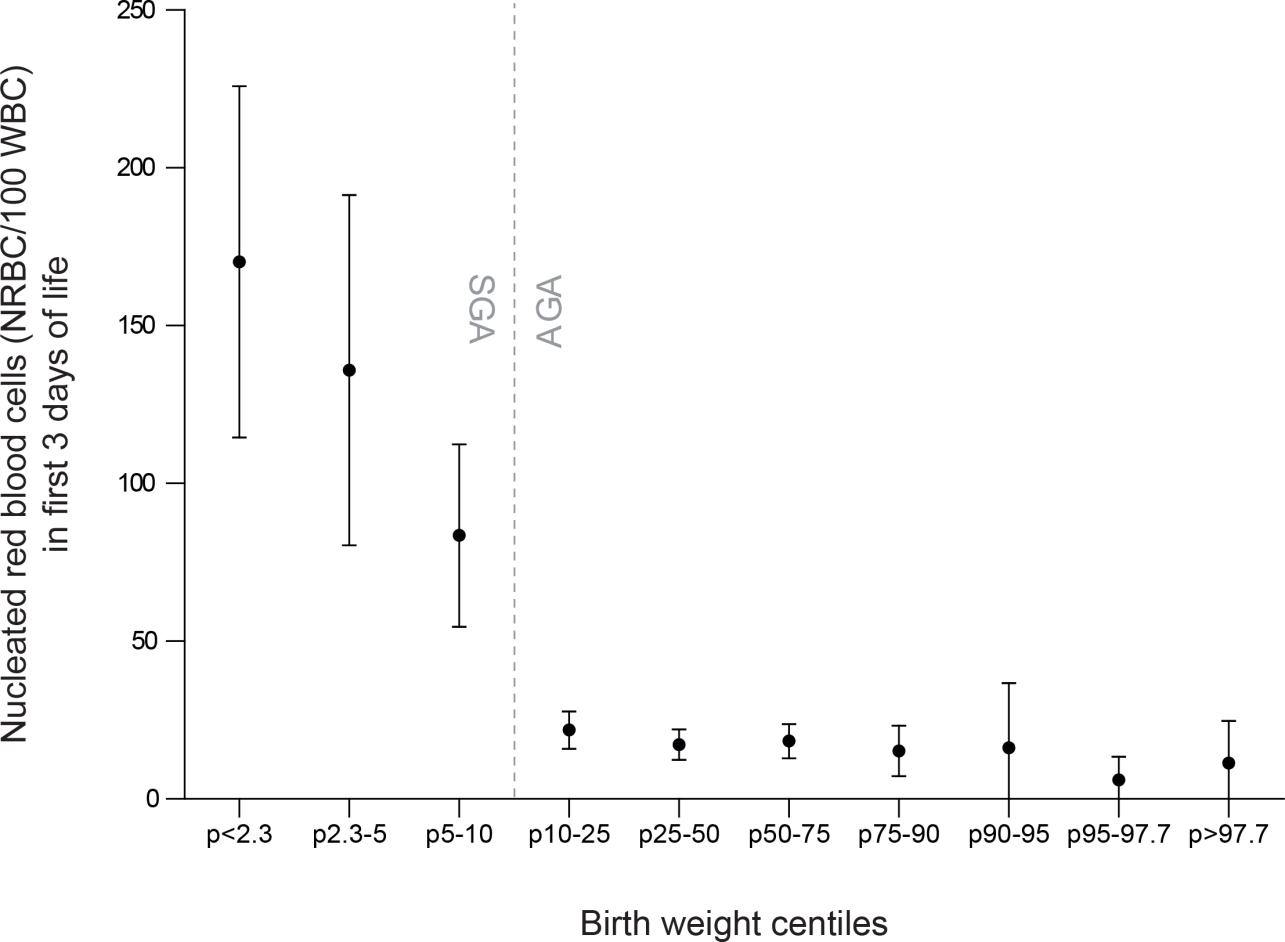
Relationship between nucleated red blood cells in the first 3 days of life and birth weight centiles. Boxplot representing observed nucleated red blood cells (NRBC/100 white blood cells) in all included neonates (SGA and AGA, N = 653) in the first 3 days of life, categorized by birth weight centiles. Boxplot shows mean (black dots) and 95% confidence intervals (whiskers). Five missing values in SGA group, 2 in AGA group.

Erythroblastosis was detected in 43% (140/325) of SGA neonates versus 10% (34/328) of AGA neonates (RR 4.2, 95% CI [3.0, 5.8]) and leucocytopenia was detected in 21% (69/329) of SGA neonates versus 6% (21/330) of AGA neonates (RR 3.3, 95% CI [2.1, 5.2]). The relationship between NRBC count and platelet count is depicted in Fig 2. Further details on hematological outcome in both groups are shown in Table 2.

**Table 2:** Hematologic outcome.

		SGA (N = 330)^$$^	AGA N = 330)^$$^	p-value
Lowest platelet count x10^9^/L*—median (IQR)		146 (96)	206 (94)	<0.001*
Thrombocytopenia—n (%) (<150x10^9^/L)		176 (53%)	66 (20%)	<0.001**
Severity of thrombocytopenia				
	Mild (100< < 150x10^9^/L—n(%)	86 (26%)	50 (15%)	
	Moderate (50< <100x10^9^/L)—(%)	65 (20%)	16 (5%)	
	Severe (20< <50x10^9^/L)—(%)	25 (8%)	2 (1%)	
	Very severe (20<x10^9^/L)—(%)	0 (0%)	0 (%)	
Proportion of neonates receiving a platelet transfusion—n (%)^$^		17 (5%)	0 (0%)	<0.001***
Hemoglobin—g/dL*		17.1 ± 3.2	17.0±2.8	0.905^##^
Nucleated red blood cells (NRBC/100 WBC)—median (IQR)		26 (106)	8 (17)	<0.001*
Erythroblastosis—n(%) #		140 (43%)	34 (10%)	<0.001**
White blood cell (WBC) count—10^9^/L*—median (IQR)		8.2 (7) ± 9.1	11.7 (8) ± 9.8	<0.001*
Leucocytopenia–n (%) (white blood cell count < 5x10^9^/L)		69 (21%)	21 (6%)	<0.001**

* Mann Whitney U Test

** Chi square test

*** Fisher’s exact test

# corrected for gestational age

^##^ student t test

^$^ None of the neonates received more than one transfusion, IQR = interquartile range.

^$$^Missing data: platelet count and white blood cell count were missing in 1 SGA neonate. Erythroblast counts were missing in 5 SGA and 2 AGA neonates.

### Bleeding outcome

Cranial ultrasound examination within the first three days of life was performed in 86% (287/330) and 80% (263/330) of SGA and AGA neonates. In the subgroup of neonates with a gestational age of <35 weeks, cranial ultrasound within the first 3 days of life was performed in 98% (203/208) and 98% (204/208) of SGA and AGA neonates, respectively. Because of the high incidence of missing ultrasounds in (near-)term infants, cerebral ultrasound outcomes are only presented for the subgroup of infants with a gestational age <35 weeks. IVH was detected in one (SGA) neonate with a gestational age >35 weeks. In one SGA neonate, the postnatal age at which the IVH occurred could not be determined. The incidence of severe IVH in the SGA and AGA groups with a gestational age <35 weeks was 1% (2/203) and 4% (9/204), respectively. This difference was not significant (Fisher’s exact test p = 0.062), but the study was not powered to assess this outcome. Of the 11 neonates with severe IVH, none had platelet counts below 50x10^9^/L. Three (27%) neonates had moderate thrombocytopenia, 3 (27%) had mild thrombocytopenia and 5 (45%) had normal platelet counts. Other possible factors that could be related to the hemorrhages included birth weight <1000 grams (n = 4), gestational age less than 28 weeks (n = 6) and respiratory distress syndrome (n = 7). Pulmonary hemorrhage occurred only in 2 neonates, both in the SGA group, one neonate had severe thrombocytopenia and the other had moderate thrombocytopenia. No cases of severe gastrointestinal hemorrhage were reported.

### Risk factors for thrombocytopenia in SGA neonates

In the subgroup of SGA neonates, four risk factors were significantly associated with platelet count in univariate analysis: low gestational age at birth, maternal hypertension, low birth weight and erythroblastosis.(Table 3). Because birth weight and gestational age are highly correlated, birth weight was excluded from the multivariable analyses. On multivariable analysis, both erythroblastosis (p<0.01) and gestational age at birth (p<0.01) remained independently associated with platelet count (r^2^: 0.32)(Table 3). The median erythroblast count in neonates with thrombocytopenia (<150x10^9^/L) was 74 (248, interquartile range) compared to 10 (20, interquartile range) in neonates without thrombocytopenia. The coefficients in Table 3 represent the number of platelets (x10^9^/L) by which a platelet count is increased (for positive coefficients) or decreased (for negative coefficients) for every unit change in risk factor, provided that the other risk factors remain constant. For example: platelet count increases with 6.7x10^9^/L with every week increase of gestational age at birth, and platelet count decreases with 43.3x10^9^/L in the presence of erythroblastosis. If the confidence interval overlaps zero, the change in platelet count is not significant. Multivariable analyses were repeated for the AGA group, in order to detect differences in risk profiles between both groups (SGA and AGA). The results were very similar, with erythroblastosis and gestational age being independent predictors of platelet count.

**Table 3:** Predictors of platelet count in neonates with SGA.

	Univariate analysis		Multivariate analysis	
	Coefficient	Confidence interval	Coefficient	Confidence interval
Maternal hypertension	-24.3	[-39.5, -9.1]*	4.6	[-9.7, 18.8]
Gestational age (per week)	8.3	[6.7, 9.9]*	6.7	[4.9, 8.5]*
Birth weight (per 100 g)	5.0	[4.0, 6.0]*	NA	NA
Moderate vs severe SGA	-12.1	[-27.1, 2.9]	NA	NA
Erythroblastosis	-59.2	[-72.8, -45.6]*	-43.3	[-56.4, -30.2]*
Early onset sepsis	-5.6	[-20.3, 9.1]	NA	NA

Coefficient: the number of platelets x 10^9^/L by which a platelet count is increased when the risk factor changes with one unit, if all other risk factors remain constant. For example: platelet count increases with 6.7x10^9^/L with every week increase of gestational age at birth, and platelet count decreases with 43.3x10^9^/L in the presence of erythroblastosis. If the confidence interval contains 0 the change in platelet count is not significant.

* significant change in platelet count. SGA = small for gestational age. Early onset sepsis = sepsis that occurred in the first 3 days of life.

## Discussion

This study confirms that early-onset thrombocytopenia is a common problem in SGA neonates, occurring in half of our SGA cohort. Our findings show that the incidence of thrombocytopenia is 2.7 times higher in SGA neonates compared to a group of AGA neonates matched for gestational age at birth, which is in agreement several other studies.[2,3,7,14,20] The strength of this study, besides being one of the largest studies on early onset thrombocytopenia in SGA neonates, is the study design with a control group matched for gestational age at birth. Matching for gestational age allowed for a more accurate comparison between the two groups (SGA and AGA) by eliminating the important confounding effect of prematurity. Our study also confirms that thrombocytopenia in SGA neonates is mild or moderate in the majority of cases (92%) and that only 5% of SGA neonates required a platelet transfusion.

As shown in Fig 1, we found a clear correlation between platelet counts and birth weight centile, emphasizing the impact of placental dysfunction and growth restriction on platelet count at birth. To our knowledge, few studies have performed this analysis in such detail before. Wasiluk *et al* found significantly lower platelet counts in severe (p<5) compared to moderate (p5-10) SGA.[10,11] Van den Hof *et al* describe a correlation between fetal abdominal circumference and fetal platelet count.[21] Because several studies have shown that reference values of platelet count in fetuses reach adult values early in gestation, it is unlikely that this correlation is confounded by gestational age.[22] Associations between platelet count and birth weight have been demonstrated before, but these birth weight groups included both AGA and SGA neonates.[4] Our results suggest that the severity of SGA, and not birth weight alone, is correlated with neonatal platelet count.

Two risk factors were independently associated with platelet count in our multivariate model. The first, erythroblastosis, was also assessed by birth weight centile, but showed a threshold pattern, with relatively constant erythroblast counts in AGA neonates, and a significant increase in erythroblast count for all SGA groups. Several other studies have also found a relationship between low platelet counts and erythroblastosis, or between SGA and erythroblasosis.[12,13,15,23–25] The cause of impaired platelet production in growth-restricted fetuses is probably related to chronic fetal hypoxia. Chronic hypoxia may induce increased erythropoietin production and erythroblastosis, which in turn may result in suppression of platelet production in the bone marrow and ensuing thrombocytopenia.[9] Murray et al have previously demonstrated that SGA neonates have an impaired megakaryocytopoiesis, shown by a marked reduction in circulating megakaryocytes.[26]

The second risk factor that was associated with platelet count in our study was gestational age, which is also in accordance with previous studies.[2,7,14] The association between gestational age and thrombocytopenia is most likely explained by maternal conditions that cause placental insufficiency, such as pre-eclampsia and pregnancy-induced hypertension. These can result in fetal growth retardation and are often managed with induced preterm delivery, which explains the relationship between thrombocytopenia and lower gestational age at birth.

Our results should be interpreted with care due to several limitations including the retrospective nature of the study and the related possibility of bias. Firstly, no distinction was made with regard to the causes of inappropriate birth weight in SGA neonates. Although SGA is mostly due to intrauterine growth restriction, some neonates may be constitutionally small. Constitutionally small infants do reach their growth potential, but are constitutionally smaller than average, in contrast to SGA neonates, who do not reach their full growth potential.[27] Constitutionally small neonates are not expected to exhibit the same hematologic abnormalities that ‘true’ SGA neonates do. Inclusion of constitutionally small neonates might therefore decrease the differences between the SGA and AGA group. Given the fact that these differences were statistically significant despite the possible mix of ‘true’ and constitutionally small SGA neonates in the SGA group, they only emphasize the impact of SGA on platelet count. Secondly, our study was not designed, nor did it have enough power to evaluate the differences in bleeding events between SGA and AGA neonates. However, a non-significant trend was observed showing no increased risk of bleeding in SGA versus AGA neonates. Several other studies have researched this question as well and suggest that bleeding risk is not increased in SGA neonates, but most of these studies were also performed in small sample sizes.[13,20,28] The fact that most neonates with major IVH did not have severe thrombocytopenia should not be surprising, since multiple studies have shown that the correlation between thrombocytopenia and bleeding risk is far from clear.[1,6,29,30] Thirdly, we did not record platelet counts after the first 3 days of life, because we focused on early onset thrombocytopenia and prenatal risk factors. However, several studies have shown that the platelet count nadir in SGA neonates occurs on day 4–5.[9,31] Because at that time not only prenatal factors, but also postnatal factors are thought to influence platelet count, identifying risk factors for thrombocytopenia after the third day of life requires additional research.

## Conclusion

In conclusion, we found that SGA neonates are at increased risk of early onset thrombocytopenia compared to AGA neonates, but that the thrombocytopenia is usually mild to moderate. A direct correlation exists between the severity of SGA and neonatal platelet count. Erythroblastosis at birth and low gestational age identify neonates at higher risk, for which monitoring of the platelet count during the first week is necessary, in order to detect severe thrombocytopenia. Further research is necessary to assess the relationship between SGA and severe bleeding.

## Supporting Information

S1 File Database. (SAV)
